# Prevalence of Body Dysmorphic Disorder Among Saudi Female Patients Seeking Cosmetic Procedures

**DOI:** 10.1192/j.eurpsy.2022.751

**Published:** 2022-09-01

**Authors:** N. Almuhanna

**Affiliations:** Imam abdulrahman bin fasial university, Psychiatry, dammam, Saudi Arabia

**Keywords:** Eastern Province, Saudi Arabia, Body Dysmorphic Disorder, cosmetic procedures

## Abstract

**Introduction:**

Body dysmorphic disorder (BDD) is a psychiatric illness in which the Patients seeking cosmetic surgery are usually unsatisfied with the outcomes of the surgery. Therefore, it is essential to study this phenomenon and increase awareness among physicians to assess for the presence of BDD before any cosmetic treatment.

**Objectives:**

To assess the presence of BDD among female patients undergoing cosmetic procedures and improve awareness among providers of cosmetic treatment.

**Methods:**

This cross-sectional study uses the adult version of the BDD modification of the Y-BOCS (BDD-YBOCS) scale. Its consists of 12 items related to preoccupied thoughts that participants have about their appearance and the effects that these thoughts have on their lives. Questionnaires were distributed on different online platforms among females living in the eastern province of Saudi Arabia.

**Results:**

Out of the 220 women who participated, 45 had BDD (prevalence rate of 20.5%), a significant and worrying percentage. The result indicates more among participants in the age group of 20–35 years. Also, it revealed positive correlation exists between BDD and females seeking cosmetic procedures.

**Conclusions:**

One-fifth of the participants were diagnosed to be suffering from BDD. Higher rates were observed among women who underwent cosmetic procedures. Therefore, we recommend physicians conduct screening for patients seeking cosmetic procedures before starting any treatment.

**Disclosure:**

No significant relationships.

